# Experimentally induced lameness in turkeys inoculated with a newly emergent turkey reovirus

**DOI:** 10.1186/s13567-015-0144-9

**Published:** 2015-02-24

**Authors:** Tamer A Sharafeldin, Sunil K Mor, Aschalew Z Bekele, Harsha Verma, Sally L Noll, Sagar M Goyal, Robert E Porter

**Affiliations:** Department of Veterinary Population Medicine and Minnesota Veterinary Diagnostic Laboratory, University of Minnesota, St. Paul, MN 55108 USA; Department of Animal Science, University of Minnesota, St. Paul, MN 55108 USA; Pathology Department, Faculty of Veterinary Medicine, Zagazig University, Zagazig, 44519 Egypt

**Keywords:** ᅟ

## Abstract

Newly emergent turkey arthritis reoviruses (TARVs) have been isolated from cases of lameness in male turkeys over 10 weeks of age. In a previous study, experimental inoculation of TARV in one-week-old turkey poults produced lymphocytic tenosynovitis at four weeks post inoculation but without causing clinical lameness. This study was undertaken to determine if TARV infection at an early age can lead to clinical lameness in birds as they age. One-week-old male turkeys were inoculated orally with a TARV (strain TARV-O’Neil) and monitored for the development of gait defects until 16 weeks of age. At 4, 8, 12 and 16 weeks of age, a subset of birds was euthanized followed by the collection of gastrocnemius tendon, digital flexor tendon, and intestines for virus detection by rRT-PCR and for histologic inflammation scoring. Clinical lameness was first displayed in TARV-infected turkeys at 8 weeks of age and ruptured gastrocnemius tendons with progressive lameness were also seen at 12–16 weeks of age. The virus was detected in gastrocnemius tendon of 4- 8- and 12-week-old turkeys but not in 16-week-old turkeys. Histologic inflammation scores of tendons at each of the four time points were significantly higher in the virus-inoculated group than in the control group (*p* < 0.01). Lesions began as lymphocytic tenosynovitis with mild synoviocyte hyperplasia at four weeks of age and progressed to fibrosis as the birds aged. These results demonstrate the potential of TARV to infect young turkeys and to produce subclinical tenosynovitis that becomes clinically demonstrable as the turkeys age.

## Introduction

Initially, turkey arthritis reoviruses (TARVs) were detected in tendons of lame, 15-week-old turkeys with swollen intertarsal (hock) joints showing histological lesions of synovial hyperplasia and inflammatory cellular infiltrates in the subsynovium [1,2]. In an effort to reproduce the disease, three different turkey reoviruses were inoculated into the footpad of 1-day-old poults but no clinical disease or histologic lesions were observed [3]. Subsequently, there were no documented reports on reovirus-induced lameness in turkeys until 2013, when we isolated TARVs from lesions of tenosynovitis/arthritis in turkeys [4]. The samples used for TARVs isolation were gastrocnemius and digital flexor tendons of 12- to 15-week-old lame turkeys.

Partial sequence analysis of the avian reovirus S4 gene showed 88.7 to 99.8% nucleotide identity between a panel of TARVs and turkey enteric reoviruses (TERVs); however, the nucleotide identity of TARVs with chicken arthritis reoviruses (CARVs) was only 78% [4]. Experimental inoculation of TARVs through oral, intratracheal, and footpad routes in 1-week-old turkey poults produced histologic lesions of marked lymphocytic tenosynovitis with synovial hyperplasia at 2 and 4 weeks post inoculation (pi). These lesions were absent in poults inoculated with TERV or CARV (5). Although TARV-inoculated birds showed high histologic scores of tenosynovitis and reovirus could be re-isolated from the gastrocnemius and digital flexor tendons, clinical lameness was not observed for up to 4-weeks pi (5 weeks of age) when the study was terminated [5].

The aims of the present work are to determine whether infection in young poults progresses to clinical lameness as they age from 1 to 16 weeks, identify the age at which lameness occurs, and determine if there is a correlation between histological tenosynovitis and clinical lameness. In addition, this work provides a detailed description of lesion progression induced by TARV in turkeys and addresses the potential of the experimental model to study TARV pathogenesis and immune response in turkeys in the future studies.

## Materials and methods

### Birds

One hundred and sixty 1-day-old male white commercial (Nicholas) turkey poults purchased from a Midwest poultry hatchery were divided into two groups (80/group) and placed in two isolation units. Meconium samples from another ten birds were collected at 1-day of age and examined for reovirus by real time reverse transcription-polymerase chain reaction (rRT-PCR) [6]. Serum samples from an additional 10 birds were tested for anti-reovirus antibodies using a commercial enzyme-linked immune sorbent assay (ELISA; IDEXX, Westbrook, ME). Birds were supplied feed and water *ad libitum*. Turkeys were supplied with a starter ration (≥30% of protein and ≤3% crude fat) until age of 8 weeks then supplied with grower/finisher ration (≥24% of protein and ≤7% of crude fat) from 8–16 weeks of age. The animal use protocol was reviewed and approved by the Institutional Animal Care and Use Committee of University of Minnesota.

### Virus

TARV-O’Neil strain of TARV isolated from leg tendons of lame turkeys in Minnesota was kindly supplied by Dr Jack Rosenberger, AviServe LLC, Newark, Delaware. The virus was grown and titrated on Japanese quail fibrosarcoma cell line (QT-35) cells and had a titer of 10^5.5^TCID_50_/mL.

### Experimental design

The poults were divided into two groups and housed separately in isolation rooms. One group of poults was orally inoculated at 1 week of age with 0.2 mL of TARV-O’Neil virus. The negative control group was inoculated with virus-free MEM. At 4, 8, 12 and 16 weeks of age, all birds were weighed and their gaits were scored using a newly developed, turkey-specific gait scoring system (Table 1). After weighing and gait scoring at each of the four time points, 10 birds from each group were euthanized by exposure to carbon dioxide gas followed by the collection of tissue samples as described below. Intertarsal (hock) joint, including distal tibiotarsus and proximal tarsometatarsus, was excised and fixed in 10% neutral-buffered formalin for histopathology. Right and left leg tendons (distal part of gastrocnemius and digital flexor tendons) and 2 cm intestinal segments (along with their digesta) were excised and stored at −20 °C for rRT-PCR. At the end of the experiment, the remaining birds (16 weeks of age) were euthanized after recording their body weights and gait scores. At each time point, Dacron swabs were inserted into the sheaths of excised gastrocnemius tendons from the ten turkeys and placed as a pool into 3 mL of brain-heart infusion broth for PCR targeting *Mycoplasma gallisepticum* and *M. synoviae*. Additionally, the gastrocnemius tendon sheaths of five birds from each treatment group were swabbed and cultured for aerobic bacteria and Mycoplasma.

**Table 1:** **Six-point (0–5) gait scoring system**

**Score**	**Description**
**0** ^**N**^	▪ Flex and extend legs smoothly and easily
	▪ No reluctance in movement
	▪ Walk actively with upright breast
	▪ Legs are parallel
	▪ Stand with no shaking in legs or labored breathing
**1** ^**N**^	▪ Flex and extend legs smoothly and easily
	▪ Little reluctance in movement
	▪ Walk actively with upright breast
	▪ Legs are parallel
	▪ Legs shake during standing
	▪ No anatomical changes in legs (swelling, valgus or varus)
**2** ^**N**^	▪ Flex and extend legs smoothly and easily
	▪ Little reluctance in movement
	▪ Walk actively with upright breast
	▪ Legs are parallel with mild swelling or had mild valgus or varus
	▪ Legs shake during standing
**3** ^**L**^	▪ Staggered movement and dropped keelbone
	▪ Unilateral significant hock joint swelling, varus or valgus
	▪ Marked bilateral defect, but can walk and stand for more than 30 seconds
**4** ^**L**^	▪ Staggered movement and dropped keel bone Marked bilateral hock joint swelling, varus or valgus
	▪ Cannot walk and stand for more than 30 seconds
**5** ^**L**^	▪ Completely recumbent
	▪ Stand and walk for seconds if it is pushed to walk
	▪ Bird then prefers to walk on hocks “creeping”
	▪ Bird may not be able to stand and creeps when initiated to move

^**N**^non-lame.

^**L**^lame.

### Gait scoring system

A six-point (0–5) scoring system was designed to score the gait of turkeys and to quantify lameness (Table 1). Each bird was observed and scored separately and any recumbent bird was gently prodded to determine if they could stand or walk independently. Turkeys with gait scores of 3 or more were categorized as clinically lame.

### Histopathology and histologic inflammation scoring

Bones comprising the intertarsal joint with adjacent gastrocnemius tendon were preserved in 10% neutral-buffered formalin. After decalcification in EDTA solution, the tissues were trimmed, processed, embedded in paraffin, sectioned at 3–4 μm, placed on glass slides, stained with hematoxylin and eosin (H&E), examined under an Olympus BX40 microscope and photomicrographs were taken by Olympus DP71 digital microscope camera. Tenosynovitis was scored histologically using a previously described histologic inflammation scoring system [5].

### Virus detection

Samples were homogenized in Hanks’ balanced salt solution (HBSS) containing 2% donor horse serum. The homogenates were then centrifuged 1500 × *g* for 20 min and the supernatant stored at −80 °C until subsequent RNA extraction and rRT-PCR. RNA extraction was done by QIAamp Viral RNA Mini Kit (Qiagen, Valencia, CA, USA). Extracted RNA was tested by rRT-PCR for reovirus S4 gene using one step RT-PCR Kit (Qiagen). This rRT-PCR showed high sensitivity and detected as few as 10 viral gene copies [6].

### Statistical analysis

The correlation coefficient between tenosynovitis (as determined histologically) and clinical lameness was calculated at 4, 8, 12 and 16 weeks of age. Detection of significant differences between the means of histologic inflammation scores and gait scores of different groups was done using the non-parametric statistical analysis “Mann Whitney U test” (NCSS 8 Statistical Software, NCSS LLC, Keysville, UT, USA). The difference between averages of body weights was tested by two sample t-test.

## Results

### Gait scoring and lameness

**Figure 1:**
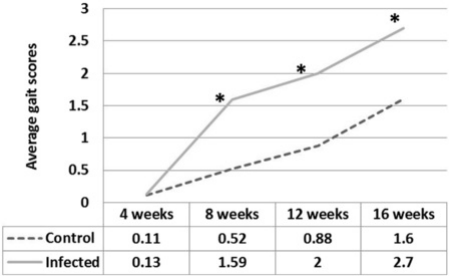
**Average gait scoring system at different time points.** The average gait scores of infected birds are significantly higher than those of non-infected controls at 8, 12 and 16 weeks of age. *Significant higher gait score at *p* < 0.05.

**Figure 2:**
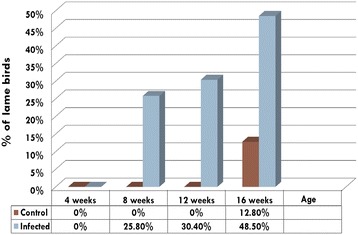
**Percentage of lame birds.** Lameness, defined as a gait score of 3 or higher, is predominant in infected birds at 8, 12 and 16 weeks of age.

**Figure 3:**
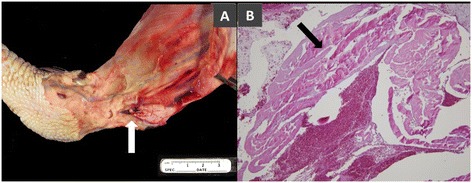
**Ruptured tendon with hemorrhage**. Tendon rupture was observed in one 16-week-old turkey. **A)** Hemorrhage at the site of ruptured tendon (white arrow). Scale bar, 1 cm. **B)** Microphotograph of shredded tendon fragment (black arrow) surrounded by erythrocytes (hemorrhage). H&E stain (40X magnification).

At the age of four-weeks, none of the turkeys in either the inoculated or the control group showed any clinical signs of lameness and their average gait score was 0. However, the gait scores in the inoculated group were significantly higher than those of control group at the ages of 8, 12 and 16 weeks (*p* < 0.05) (Figure 1). Clinical lameness (gait score >3) was observed at 8 weeks of age and progressively increased as the inoculated birds aged; clinical lameness was observed in 26%, 30%, and 48% of inoculated birds at 8, 12, and 16 weeks of age, respectively. There was a minimal increase in gait scores in turkeys of the control group from 4 to 12 weeks of age but 5 of 39 birds (13%) showed lameness at 16 weeks (Figure 2). Virus-inoculated turkeys showed swollen hock joints with breast blisters and breast buttons with a single bird showing ruptured gastrocnemius tendon at 16 weeks (Figure 3).

### Histologic inflammation scores

**Figure 4:**
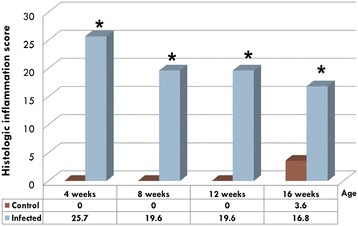
**Means of histologic inflammation scores in gastrocnemius tendon sheath.** Infected birds had significantly higher averages of histologic inflammation scores at 4, 8, 12 and 16 weeks of age.*Significantly higher score (*p* < 0.01).

**Figure 5:**
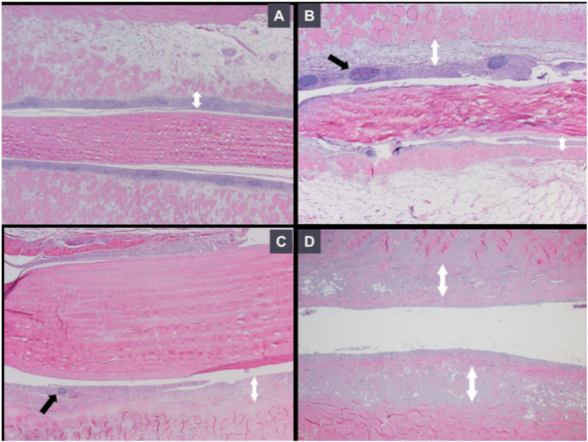
**Lesion progression.** Microphotographs show the progression of inflammation in gastrocnemius tendon sheath of 4- to 16-week-old infected turkeys. **A)** Prominant lymphocytic infiltration (White up-down arrow) (4-week-old); **B)** Lymphocytic nodules (Black arrow) present and fibroplasia (White up-down arrow) starts (8-week-old); **C)** Fibrosis increases (White up-down arrow) and lymphocytic infiltration (Black arrow) decreases (12-week-old); **D)** Fibrosis (White up-down arrow) is prominent (16-week-old). H&E stain (40X magnification).

Mean histologic inflammation scores of gastrocnemius tendon sheaths were significantly higher (*p* < 0.01) in the inoculated group as compared to the control group at all ages (4, 8, 12 and 16-weeks) (Figure 4). The lesions consisted of prominent lymphocytic infiltration of the subsynovium and mild synoviocyte hyperplasia at 4 and 8 weeks, along with mild fibroplasia and lymphoid nodule formation at 8 weeks, progressing to decreased lymphocytic infiltration and increasing fibrosis at 12 weeks, and finally a prominent subsynovial fibrosis at 16 weeks (Figure 5).

### Correlation between tenosynovitis and gait score

**Figure 6:**
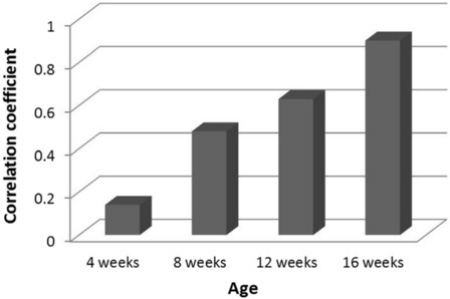
**Correlation coefficient between gastrocnemius tendon histologic inflammation score and gait score.** The correlation is low at 4 weeks of age as no birds showed clinical lameness though they had high histologic inflammation scores. The correlation gradually increased to 0.9 at 16 weeks of age.

The gait score and histologic inflammation score for each bird were used to calculate correlation coefficient, which was low (0.1) at 4 weeks and progressively increased until a strong positive correlation (0.9) was observed between tenosynovitis and high gait score (lameness) at 16 weeks (Figure 6).

### Body weight

**Figure 7:**
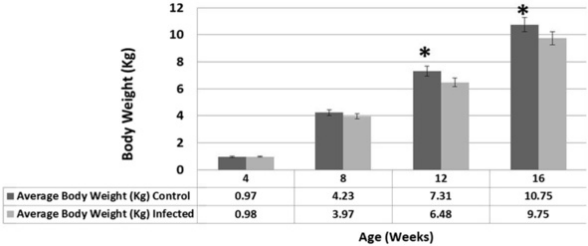
**Average body weights at different time points.** At 12 and 16 weeks of age, infected birds had a significantly lower average body weight compared with non-infected controls. *Significant higher body weight (*p* < 0.05).

The average body weight of inoculated birds was significantly lower (*p* < 0.05) than that of control birds at 12 and 16 weeks (Figure 7).

### Virus detection

Fecal and serum samples taken from 1-day-old poults were negative for reovirus and antireovirus antibodies, respectively. In addition, samples from 1, 4, 8, 12 and 16-week-old turkeys were negative for aerobic bacteria by culture and negative for *Mycoplasma synoviae* and *M. gallisepticum* by culture and PCR. Reovirus was detected by rRT-PCR in 4 of 10 tendon and 3 of 10 intestine samples of 4-week-old turkeys in the virus-inoculated group. In 8-week-old birds, 5 of 10 tendons and 4 of 10 intestines were positive while at 12 weeks, only 2 of 10 tendon and 2 of 10 intestinal samples were positive for reovirus by rRT-PCR. All tissues collected from negative control turkeys at all ages were negative for reovirus by rRT-PCR.

## Discussion

Previous work in our laboratory showed that poults orally inoculated with TARV at 1-week of age developed lymphocytic tenosynovitis by 4 weeks pi but did not show clinical lameness [5]. Our present study sought to determine if tenosynovitis induced by TARV would progress to clinical lameness as the birds aged, to characterize the lameness through gait scoring, and to determine the relationship between tenosynovitis and gait scores. The study also described the progression of histologic lesions from ages 4 to 16 weeks. The study aimed in general to test the potential of the experimental model for future experimental studies.

Lymphocytic infiltration of the gastrocnemius tendon sheath was prominent in 4-week-old turkeys and progressively decreased by the time the turkeys were 16 weeks of age, while subsynovial fibrosis was initially observed in 8-week-old turkeys and was most prominent in 16-week-old turkeys. These results resemble those shown in a long term study on chicken reoviral arthritis [7], in which oral inoculation of 1-day-old chicks with chicken reovirus led to the development of gastrocnemius lymphocytic tenosynovitis and fibroplasia by 10 weeks of age and predominant fibrosis by 20 weeks of age.

The fibrosis of tendon and tendon sheath might lead to a decrease in the tensile strength of tendons as the birds age and become heavier, especially in domestic turkeys that have a high growth rate. In addition, tendon adhesion with heavy weight as a contributing factor might have led to tendon rupture [8]. Similar results were shown in chickens infected with chicken arthritis reovirus; at 10 weeks pi there was fibrosis and adhesion of the gastrocnemius tendon, which along with the stress of heavier body weight resulted in tendon failure and rupture [9].

Lameness evaluation in this work required a gait scoring system that includes all variations of gait abnormalities. For that reason, a previous 4-point gait scoring system in turkeys [10] did not suit our requirements. The previously designed 6-point (0–5) gait scoring system designed for chickens [11] and its modifications [12] were also not suitable because of behavioral variation between chickens and turkeys. Our newly designed 6-point (0–5) gait scoring system is specific for turkeys in which scores of 3 and above are considered as clinically lame. There was a clear distinction between score 2 and score 3 to avoid confusion and to have a clear cut point that distinguishes between lame and non-lame birds. The average gait score of each infected group was not less than 3, but was significantly higher than the average score of the corresponding control group.

Clinical lameness was first observed in 8-week-old turkeys with 25.8% of inoculated turkeys being lame. The percentage of lame birds in the inoculated group progressively increased at 12 weeks (30.4%) and 16 weeks (48.5%) while negative control turkeys, for the most part, remained clinically normal. The correlation coefficient between tenosynovitis score and degree of lameness was low (0.1) in 4-week-old turkeys; these birds had high tendon inflammation scores (tenosynovitis) but did not show clinical lameness. These findings were in agreement with our previous work that showed no clinical lameness in 5-week-old turkeys that had been orally inoculated with TARV at 1 week of age, despite the presence of high gastrocnemius tendon inflammation scores [5]. In the present study, the correlation between tenosynovitis and gait score increased dramatically from 8 to 16-week-old turkeys with a peak of 0.9 at 16 weeks. This high correlation coefficient was attributed to increased clinical lameness in tenosynovitis birds and this was associated with increased body weight (Figure 6). Studies on reovirus infection in chickens have demonstrated a strong association of lameness with fibrosis of the gastrocnemius tendon and tendon sheath, tendon adhesion and eventual rupture as the chickens increased in body weight [9]. In addition, it has been shown that the greater weight gain in meat producing chickens might affect the physical consistency of tendons during reovirus infection leading to clinical lameness [13].

In control birds at 16 weeks of age there were 5 lame birds out of 39. These birds were negative for reovirus, bacteria and mycoplasma and they did not show tenosynovitis. A possible noninfectious cause of lameness might be the cause. Tibial dyschondroplasia (TD) which is a genetic disorder in which cartilage at bone epiphysis fails to ossify leading to weak legs and lame birds [14]. When the proximal epiphysis of tibia is cut in necropsy cartilage mass will be seen in epiphysis [15] which we did not do in 16-week-old turkeys. It is possible that it was a valgus–varus deformity which was associated with overgrowth rate and may show gastrocnemius tendon slippage [16]. Perosis and chondrodystrophy may be induced in vitamin B complex and mineral deficiency [16-18] which we think that it was not in our turkeys. We have not found any footpad ulcers and litter was kept dry and clean all the time but this might be a shaky leg lameness which occurs mostly in male turkeys 8–18 weeks of age due to low quality moist litter [19]. TARV-infected turkeys had significantly lower average body weights than control groups at 12 and 16 weeks of age. Because reduced body weights were observed when gait scores started to increase at 8 weeks, we suspect that the discomfort associated with lameness made it more difficult for the affected turkeys to reach the feeders and likely reduced feed consumption.

In a previous study, two-day-old turkeys inoculated with enteric reoviruses showed significantly lower body weight than controls at 2 to 9 days pi [20]. This might be due to the age of inoculation since younger birds have been shown to be most susceptible to reovirus infection. In our study, we inoculated turkeys at 1 week of age. The effect of age of inoculation on TARV infection has not yet been studied in turkeys but this age susceptibility has been demonstrated in chickens challenged with reovirus [21]. TARV not only affects the carcass quality (e.g., hock swelling, breast blisters) of turkeys but, under experimental conditions also results in a significantly lower body weight at market age (Figure 7).

In this study, virus was detected by rRT-PCR in 20%-50% of tendon and intestinal samples in infected turkeys at 4, 8 and 12 weeks of age with the peak of virus detection in 8-week-old turkeys. At 8 and 12 weeks of age, all rRT-PCR positive birds had lameness gait scores (3 or more). In field cases, we originally diagnosed tenosynovitis and successfully isolated reovirus from tendons of 16-week-old turkeys, but under experimental conditions we were not able to detect reovirus in tendons of 16-week-old turkeys by using rRT-PCR, which was previously shown to be of higher sensitivity in TARV detection than virus isolation [6]. Reovirus has been detected in field cases of chickens with tenosynovitis/arthritis at 11 weeks of age and younger [22]. It appears that experimental conditions are different from field conditions, perhaps in terms of overall viral load and viral shedding and cycling in field turkeys. In addition, although we do not know when field turkeys are naturally infected with TARV, our work indicates that surveillance of turkeys for TARV should occur weeks before any anticipated lameness occurs (mostly observed in field cases >12-week-old) in order to increase detection of virus, particularly if field poults are naturally infected vertically through the egg or at a very young age, as has been demonstrated in chickens [23]. In conclusion, infection of poults with TARV causes lymphocytic gastrocnemius tenosynovitis that over time progresses to subsynovial fibroplasia and fibrosis. These changes along with increased body weight lead to clinical lameness and occasional tendon rupture as the birds age. In addition, TARV is most readily detected in infected turkeys prior to or as soon as the lameness is observed. We developed a gait-scoring system that should be useful for veterinarians or production personnel to characterize early alterations in gait, thus identify earlier onset of TARV infection and increase the opportunity for detection of TARV in infected turkeys. These findings augment the similarity of the experimental model (inoculation of TARV-O’Neil orally at 1-week-old) with the field condition and prove the potentiality of using this model in future immunopathogenesis studies.
